# Cost-benefit analysis of alternative tax policies on sugar-sweetened beverages in Mexico

**DOI:** 10.1371/journal.pone.0292276

**Published:** 2023-10-03

**Authors:** Juan Carlos Salgado Hernandez, Shu Wen Ng, Sally C. Stearns, Justin G. Trogdon

**Affiliations:** 1 National Institute of Public Health, National Council of Humanities, Science and Technology, Mexico and Center for Research in Nutrition and Health, Cuernavaca, Mexico; 2 Carolina Population Center and Department of Nutrition, Gillings School of Global Public Health, University of North Carolina at Chapel Hill, Chapel Hill, North Carolina, United States of America; 3 Department of Health Policy and Management, Gillings School of Global Public Health, University of North Carolina at Chapel Hill, Chapel Hill, North Carolina, United States of America; Wuhan University, CHINA

## Abstract

In 2014, Mexico implemented a tax on sugar-sweetened beverages (SSB) equivalent to one Mexican peso (MP) per liter to address the high obesity prevalence. This tax has effectively reduced SSB purchases and yielded healthcare savings; however, it remains unknown whether SSB taxes lead to net benefits at the societal level in Mexico. Moreover, public health experts recommend increasing the tax. The objective of this study is to estimate the net benefits of SSB taxes compared to a scenario of no tax in urban Mexico. Taxes include the one-MP tax and alternative higher taxes (two and three MP per SSB liter). Thus, we conducted a cost-benefit analysis from the perspective of the government, producers, and consumers for a simulated closed cohort of adults in a life-table model. We defined net benefits as the difference between economic benefits (the value of statistical life, healthcare savings, and tax revenue) and costs (consumer surplus and profit losses). We found that, at the societal level, all simulated taxes will eventually generate benefits that surpass costs within ten years. Overall net benefits can reach USD 7.1 billion and 15.3 billion for the one-MP and the three-MP tax, respectively. Hence, these benefits increased at a declining rate compared to taxes. The government and consumers will experience overall positive net benefits among society’s members. Policymakers should consider time horizons and tradeoffs between health gains and economic outcomes across different society members.

## Introduction

In January 2014, the Mexican federal government implemented a specific tax of one Mexican peso (MP) per liter of sugar-sweetened beverage (SSB) to address the high prevalence of overweight and obesity. Evidence showed SSB price increases coupled with SSB purchase reductions after the tax implementation [1,2]. Moreover, the one-MP SSB tax is expected to yield population health improvements and healthcare savings for the Mexican government [3,4]. The government is expected to also benefit from the money raised via tax revenue.

The positive effect on public finances and health outcomes may make the SSB tax an appealing health policy. However, three SSB-tax-related costs should also be considered. First, the government will face tax implementation and monitoring costs while also funding additional social security needs due to a life expectancy increase linked to the tax. Second, SSB producers might lose profit due to purchase reductions or by absorbing the tax partially. Third, consumers will experience a welfare loss from the displeasure of devoting more money to get an SSB or the lost enjoyment when decreasing their SSB consumption (i.e., reduction in consumer surplus). Thus, consumers will be deprived of SSB attributes that they value, such as sugar or fizz.

Several studies have included economic benefits and costs in their SSB-tax economic assessments. In Mexico, the SSB tax is expected to generate healthcare savings equivalent to USD 4 per dollar spent on operating costs of the SSB tax by the government [4]. A systematic review found SSB taxes were a cost-saving intervention [5]. Studies in the United States and the United Kingdom have taken a more comprehensive scope by including the SSB tax impact on producer profits and/or consumer surplus in addition to healthcare costs [6,7]. These studies found that SSB taxes would yield social gains due to healthcare savings and internality corrections across consumers (e.g., mitigation of self-control problems) outweigh consumer or producer losses.

In Mexico, no study has assessed the joint SBB tax effect on consumers, producers, and the government. This study aims to fill this research gap based on a cost-benefit analysis (CBA) of the current one-MP SSB tax and other SSB tax policies in urban Mexico. The alternative policies include a tax of two MP per SSB liter recommended by public health experts [8] and an additional increase to reach three MP per SSB liter. We build our CBA upon previously published models: a structural model of demand and supply for the Mexican non-dairy and non-alcoholic beverage market and a life table epidemiological model [2,9]. The comprehensive evaluation of SSB taxes can inform policymakers in different dimensions when evaluating SSB taxes in Mexico. Specifically, we provide evidence on how different SSB tax policies impact health outcomes and how their economic consequences for society evolve over time.

## Materials and methods

### Data

The main information source for the structural model for the Mexican non-dairy and non-alcoholic beverage market is the Nielsen Mexico Consumer Panel Service (Nielsen CPS) from 2012 to 2015 [10]. These data contain information on household purchases for pre-packaged foods and beverages [10]. Nielsen CPS is representative of cities with >50,000 inhabitants, representing 63% of the Mexican population and 75% of the national food and beverage expenditure in 2014 [10,11].

For the epidemiological model, we used pre-tax data (i.e., before 2014). Information on Mexicans’ weight and height and SSB consumption by age group and sex in urban areas (>2,500 inhabitants) came from the 2012 National Health and Nutrition Survey (ENSANUT in Spanish) [12]. We derive information on prevalence, incidence, and case fatality at the disease level in Mexico in 2013 based on the Global Burden of Disease (GBD) study and overall mortality in 2013 from the Mexican National Population Council (CONAPO in Spanish) [13,14]. Specifically for disease-specific information using GBD data, we calculated prevalence as the ratio between cases of the relevant disease and the overall relevant population, incidence as the ratio between new cases of the relevant disease and susceptible population (i.e., subtracting existing cases from the overall relevant population), and case fatality as the ratio between deaths and cases of the relevant disease. We calculated all these measures by age group and sex in 2013. The GBD study lacked information on the incidence of hypertensive heart disease, and we estimated it using DISMOD II [15]. Population size by sex and age at the beginning of 2014 came from CONAPO [14]. We used a population size equivalent to 63% of the national population aged ≥ 20 years, in line with the representativeness of Nielsen CPS and the epidemiological model explained below.

We retrieved average information on the annual healthcare cost per patient from the Mexican Ministry of Health for most diseases [16]. Healthcare cost information for diabetes by age group came from Sanchez-Romero et al. [3]. We assume that the average cost for diabetes among people aged 20–54 years in our CBA was the average for age groups of 35–44 and 45–54 years in the study by Sanchez-Romero et al. [3] because this study had no cost information for people younger than 35 years. Due to the lack of information on kidney cancer costs, we derived it from a disease cost ratio. We informed this ratio based on cost information from Ecuador and Chile based on an analysis by Fernandez et al. [17]. These countries are likely to share common characteristics with Mexico due to all being in Latin America. Specifically, we assumed that the ratio of the annual cost per patient of kidney cancer to colon cancer in Mexico was the average of this ratio in Ecuador and Chile [17].

In our study, the economic consequences linked to the tax effect on mortality are composed of two major components. First, the government will have to fund public retirement pensions and healthcare utilization for people who would have died in the absence of the tax. We retrieved information on the average public retirement pensions for people aged ≥ 65 years and the probability of getting this pension from the National Expenditure and Income Survey [18]. Moreover, we calculated the costs of the additional healthcare utilization using the information on the public health spending not allocated to treat obesity-related diseases and how the average per capita healthcare cost varies as people age. Second, to account for the economic valuation by consumers linked to mortality reductions after the tax implementation, we used the value of a statistical life (VSL). VSL corresponds to people’s willingness to pay to reduce their mortality risk and is supposed to capture the monetary and non-monetary aspects that people attach to this reduction in mortality risk [19,20]. We used the VSL estimate from the Mexico City metropolitan area (i.e., USD 280,743; 2014 value) [21].

We complemented the economic data using the information on wages for people with the right to paid sick days and disability pensions from the Mexican Ministry of Health and information on the percentage of people working in Mexico from the Mexican National Occupation and Employment Survey [16,22]. We calculated the average caregiving time by patients’ relatives by disease and sex group using information for Mexico from the Economic Commission for Latin America and the Caribbean [17]. We translate this time into monetary value based on the average hourly minimum wage in Mexico in 2014 [23], from which we assumed an average working day of eight hours. S1.1 Table in S1 File lists the reference of all the above-mentioned inputs. In S1.2 Table in S1 File, we report the summary statistics of the main model input information. In S1 Metadata in S1 File, we present the disaggregated economic information in S1.2 Table in S1 File by sex-age group (when applicable). Moreover, this metadata includes epidemiological information on population size and overall mortality, prevalence, incidence, case fatality by disease of interest, body mass index (BMI) and variability in SSB consumption.

### Structural model

The structural model of demand and supply for the Mexican non-dairy and non-alcoholic beverage market allows us to assess the effect of different SSB tax policies in urban Mexico [2]. This market is composed of untaxed (i.e., water and diet SSB) and taxed beverages (i.e., SSB). The demand model is built upon the assumption that consumers purchase the taxed or untaxed beverage that provides the highest level of satisfaction (known as utility in economics) according to their preferences over products’ attributes with no health-related concerns. Below, we relax this assumption by allowing some degree of health awareness. The demand model corresponds to a variety of a logit model (i.e., random-coefficients logit model) whose functional form entails that the demand does not hold a linear relationship with prices. For the empirical application of the model for the beverage market in Mexico, the authors modeled households’ preference over sugar content and prices while accounting for brand-fixed effects in the utility function [2]. For the supply model, the authors of the structural model assumed that producers compete in a Bertrand-Nash supply model and set prices that guarantee the highest profits across their product portfolio, including taxed and untaxed beverages. In practice, the supply-related part of the structural model is recovered by using the first-order condition under which producers are theoretically supposed to maximize profit when setting prices.

When an SSB tax comes into effect, the structural model accounts for the simultaneous response by consumers (seeking to maximize utility) and producers (seeking to maximize profits) that results in new equilibrium information on prices, purchases, tax revenue, consumer surplus, and profit. Derived changes from the model in consumer surplus and profit capture offsetting gains arising from the substitution between taxed and untaxed beverages in response to price increases after the tax implementation. We used the structural model to estimate all these outcomes for the three tax levels of interest (i.e., $1 MP, $2 MP, and $3 MP). Appendix A2 in S1 File shows equations to calculate the tax impact on relevant outcomes. S1.3 Table in S1 File presents the summary statistics of the data used in the structural model. This table shows price and purchase information from Nielsen CPS [10] before (i.e., 2012–2013) and after (i.e., 2014–2015) the tax implementation.

### Epidemiological model

We estimated the SSB tax effect on health outcomes using a published epidemiological life-table model by Veerman et al. [9]. This model simulates health-related trajectories for the reference population and the intervention population. For the former population, trajectories are based on life tables that depend on sex-age groups’ information on overall mortality, the no-intervention-affected BMI, and linked mortality and morbidity for a set of diseases. In a similar fashion, the model estimates the health-related trajectories for the intervention population but accounting for the calorie reduction due to the intervention of interest. This calorie reduction will lead to a body weight loss following Hall et al., who posited that a permanent daily change of 10 calories would generate a steady-state change in adults’ weight of one pound [24]. The body weight loss will reduce the population’s BMI and thus the mortality and morbidity for a set of diseases according to the potential impact fraction. This fraction captures reductions in disease-specific risks of incidence in response to reductions in a risk factor [25], which corresponds to BMI in the model. The model allows the population’s members to develop multiple diseases. These diseases correspond to diabetes, ischemic heart disease, stroke, hypertensive heart disease, osteoarthritis, and a set of cancers (i.e., breast, colon, and kidney).

The intervention effect arises from the difference in outcomes between the reference population and the intervention population. The epidemiological model provides yearly estimates by sex for adults aged ≥20 years from the intervention implementation until people turn 95 years old or die (split into 15 five-year age groups). Hence, the model represents a closed cohort that gets smaller over time. Below, we present the results for different time horizons.

For the empirical model, we assumed a normal distribution for SSB purchases and a lognormal distribution for BMI and relative risks of incidence at the disease level. Moreover, we assumed that as people age, their average BMI would be the average for the next age group, which entails an increasing trend for several age groups (see S1.4 Table in S1 File). Due to the structural model provides a point estimate of SSB purchases, we allowed these purchases to resemble the variation in the SSB consumption by age group and sex in urban Mexico as in the 2012 ENSANUT [12]. We present the SSB consumption distribution by sex-age group in S1.5 Table in S1 File.

We set 2014 as the year of the SSB tax implementation. We run the model using Excel and the add-in by Epigear software [26]. This software allows us to estimate the potential impact fraction and to account for the uncertainty from the tax effect on BMI and disease-specific relative risks. We conducted a Monte Carlo simulation for each tax policy based on 2,000 iterations to account for the model’s uncertainty. We report their average and 95% uncertainty ranges based on these iterations.

### Cost-benefit analysis

The SSB-tax economic benefits for the government include healthcare savings, fewer disability pensions and paid sick days, and tax revenue. However, the government will face costs by funding public retirement pensions and healthcare services unrelated to overweight or obesity due to the survival increase after the tax implementation. See Appendix A3 in S1 File for the procedure to estimate the cost of the latter healthcare services.

We decompose the tax effect into three components for society’s members other than the government. The first component is the costs arising from reductions in producers’ profit and consumer surplus due to SSB purchase decreases attributable to the tax. Second, due to fewer disease cases after the tax implementation, consumers’ relatives will benefit by allocating less time to serve as caregivers, which we translate into monetary value according to the average hourly minimum wage. Third, we transform the mortality reduction into economic benefits for consumers based on VSL.

Under a conservative scenario, there would be no need to account for VSL benefits by assuming consumers are fully informed and rational and thus fully internalize any adverse health effect when purchasing SSB. Hence, consumer surplus would reflect this internalization. Conversely, under an optimistic scenario, consumers would experience the full VSL benefit when completely lacking information on SSB-related adverse health effects. Due to the infeasibility of these extreme scenarios, we set the economic benefit to be equivalent to 80% of the VSL per avoided death under the assumption that consumers internalize 20% of the negative SSB effect on health, which entails it is already part of the consumer surplus. This 20% is close to the expected proportion of around 22% of Mexican adults who use information from front-of-package labels (for the label design preceding the warning labels implemented in October 2020) and distinguish an unhealthy ultra-processed product (i.e., high in nutrients linked to poor health outcomes such as sugar and sodium) according to these labels. Specifically, 36% of Mexican adults reported sometimes or usually using front-of-package labels, and 61.5% of Mexican adults classified an unhealthy ultra-processed product as unhealthy or partially unhealthy based on these labels (36% x 61.5% ≈22%) [27].

The SSB tax net benefit arises from the difference between costs and benefits described above. In sensitivity analyses, we assume no VSL benefits, which implies an internalization rate of 100% when purchasing SSB in line with the conservative scenario described above. Conversely, the optimistic scenario corresponds to full benefits from VSL equivalent to an internalization rate of 0%, which we also include as part of the sensitivity analyses.

In light of the relevant stakeholders described above, our CBA is from the perspective of the government, producers, and consumers. We transformed all economic information for our CBA into 2014 values using the Mexican consumer price index [28]. We report all our economic results in USD based on the exchange rate in 2014 and a discount rate of 4%. This discount rate corresponds to the recommended rate for an upper-middle-income country like Mexico [29]. In Appendix A4 in S1 File, we explain how we calculate each CBA component.

## Results

Table 1 shows outcomes from the structural model. Compared to a simulated counterfactual with no tax, SSB price increases are larger than the tax amount by around 20%. These results are in the range of previous studies in Mexico assessing price changes under the one-MP tax, which include tax over-shifting (i.e., price increase above the tax amount) [30]. Likewise, ex-ante evaluations of the SSB tax have shown tax over-shifting for per-unit taxes [31], which is the kind of SSB tax in Mexico. Meanwhile, SSB purchase reductions are proportionally lower than SSB tax increases because there is a no-linear relationship between demand and prices in the structural model. For example, when doubling the tax from one MP to two MP, SSB purchases decreased from 18.97% to 34.08%. This result could arise from consumers displaying a decreasing marginal utility (i.e., the lower the quantity the consumers hold, the less they are willing to reduce their consumption due to higher utility reductions) and a decrease in the extent of tax over-shifting across tax rates.

**Table 1:** Results of the structural model for sugar-sweetened beverage (SSB) taxes.

Outcome	No tax	Tax effect		
		No tax vs $1.0 MP tax	No tax vs $2.0 MP tax	No tax vs $3.0 MP tax
SSB Prices ($ MP)	8.17	1.24	2.38	3.52
SSB Purchases (%)	—	-18.97	-34.08	-46.77
PC-PD SSB Purchases (ml)	204.14	-38.72	-69.58	-95.51
(Consumer surplus and profit loss)/Tax revenue (MP)	—	-1.45	-1.64	-1.86
(Consumer surplus loss)/Tax revenue (%)	—	60.25	58.18	56.97
(Profit loss)/Tax revenue (%)	—	39.75	41.82	43.03

Source/Note: SOURCE. Authors’ analyses based on a structural model and data from Nielsen through its Mexico Consumer Panel Service (CPS) for the food and beverage categories for January 2012—December 2015.[2,10] NOTES. SSB: Sugar-sweetened beverages. PC-PD: Per capita per day. MP: Mexican pesos. The Nielsen Company, 2016. Nielsen is not responsible for and had no role in preparing the results reported herein.

Even though we do not report the price elasticities of demand in Table 1, we can approximate them by dividing the percentage quantity change by the percentage price change. This will entail an elasticity between -1.25 and -1.10, which is in the range of previous estimates in Mexico [30]. Table 1 also shows that consumer surplus and profit reductions get larger with SSB tax increases. These reductions are $1.45 and $1.86 per MP collected as tax revenue for the one-MP tax and the three-MP tax, respectively. The reduction is more concentrated among consumers (i.e., 57–60%) than producers (40–43%).

**Fig 1:**
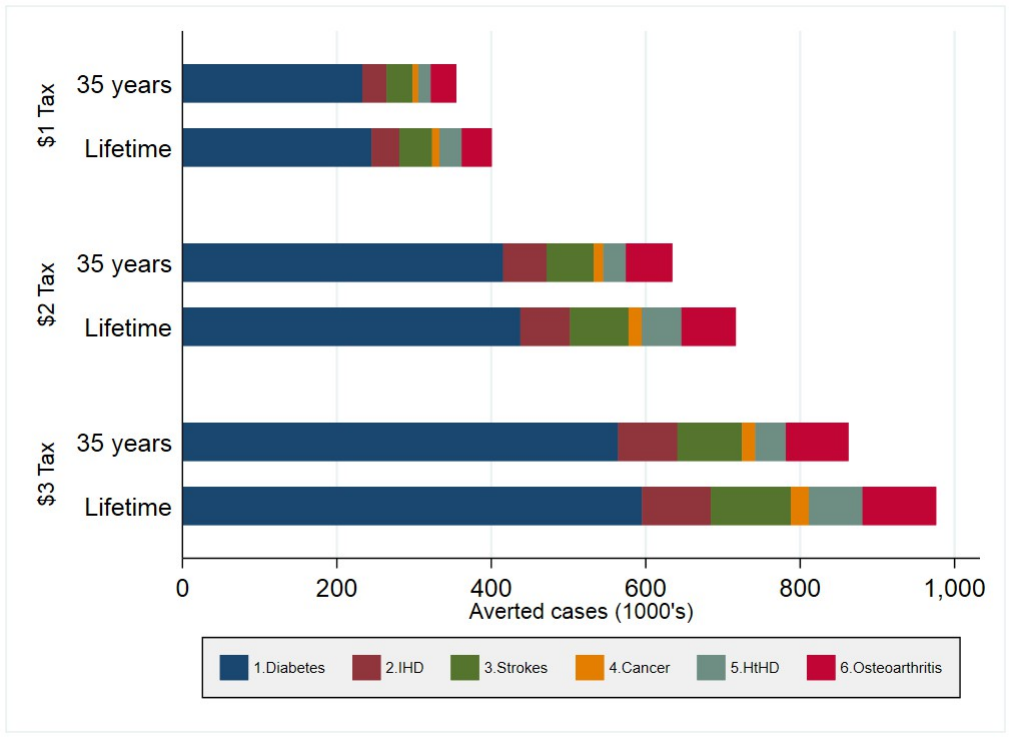
Cumulative avoided new incident cases by SSB tax policy. Source/Note: SOURCE. Authors’ analyses based on structural models and an epidemiological model and inputs in Table A1.1. [2,9] NOTES: IHD: ischemic heart disease; HtDH: hypertensive heart disease; and cancer represents a set of cancers (breast cancer, colon cancer, and kidney cancer).

**Fig 2:**
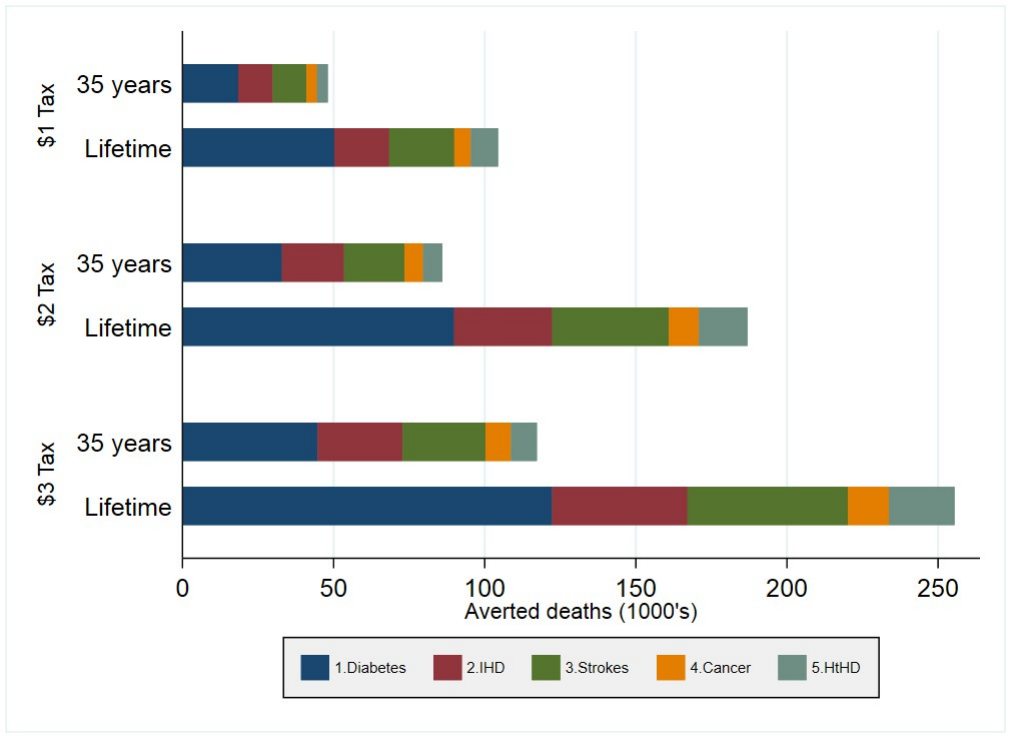
Cumulative avoided deaths by SSB tax policy. Source/Note: SOURCE. Authors’ analyses based on structural models and an epidemiological model and inputs in Table A1.1. [2,9] NOTES: IHD: ischemic heart disease; HtDH: hypertensive heart disease; and cancer represents a set of cancers (breast cancer, colon cancer, and kidney cancer).

Figs 1 and 2 present the SSB tax effect on the cumulative reduction of disease-specific incident cases and deaths, respectively. The different SSB tax policies are likely to reach most of their effect for the new incident cases after 35 years. Conversely, the SSB tax effect on mortality is expected to be larger during the last years of the cohort. Diabetes and ischemic heart disease concentrate most of the population’s health improvements. S4.1 and S4.2 Tables in S1 File include health outcomes with their uncertainty ranges.

**Fig 3:**
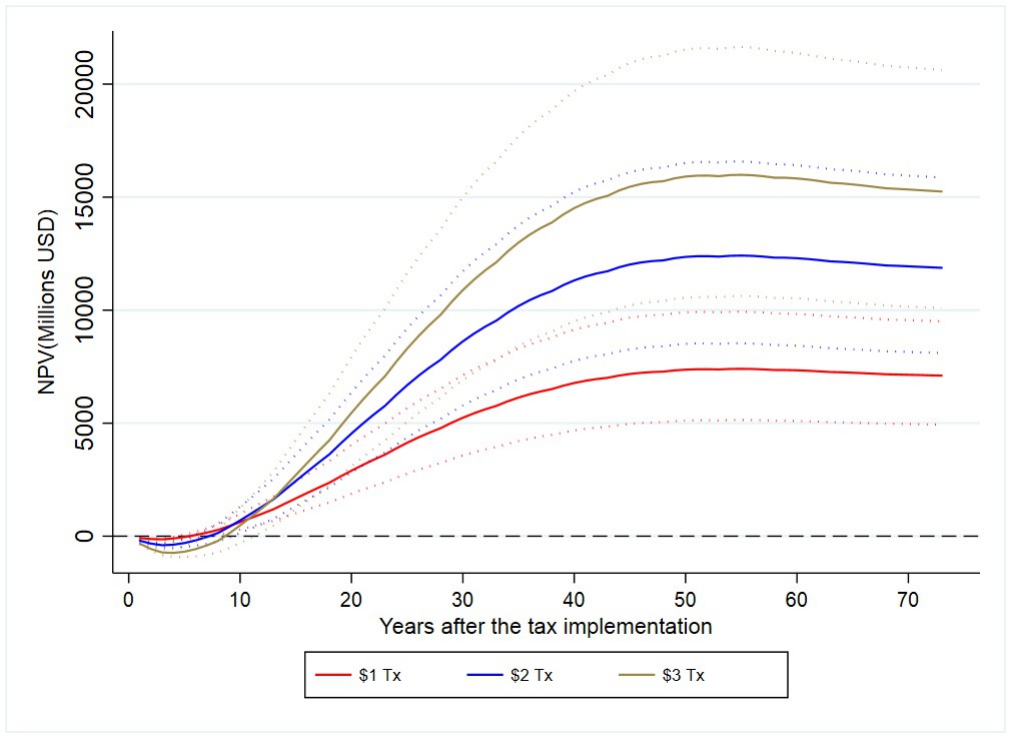
Cumulative discounted net benefit by SSB tax policy. Source/Note: SOURCE: Authors’ analyses based on structural models and an epidemiological model and inputs in Table A1.1.[2,9] NOTES: Information in 2014 Dollars and based on a discount rate of 4%. NPV stands for net present value. Sensitivity ranges (95%) in dotted lines.

Fig 3 presents the yearly cumulative discounted net benefit by SSB tax. During the first years under any tax policy, cumulative costs surpass cumulative benefits, which results in negative net costs. These net costs tend to get larger and prevail longer when the SSB tax is higher. The break-even point occurs as early as six years under the one-MP SSB tax or as late as nine years under the three-MP SSB tax. Thus, all SSB tax policies are predicted to yield positive health benefits beyond ten years. Even though the positive net benefits get larger when the tax amount goes up, this increase is proportionally lower than the increase in the tax. For the cohort’s lifetime, the one-MP SSB tax will yield cumulative discounted net benefits close to USD 7.1 billion, while this number is USD 15.3 billion for the three-MP SSB tax.

In Table 2, we decompose the SSB tax net benefit into benefits and costs by society’s members. Regardless of the tax policy or timespan, benefits are always larger than costs for the government. The main components of these benefits are tax revenue and healthcare savings. For consumers across tax policies, costs in terms of consumer surplus losses surpass benefits after ten years of the tax implementation. However, this pattern is reversed over time (i.e., within 35 years of the tax implementation), with VSL gains as the main driver. Finally, and as expected, producers only experience costs as profit reductions.

**Table 2:** Discounted costs and benefits (Million USD) by SSB tax policy and society’s member.

	Government				Consumers			Producers	Overall society
	Benefits			Costs	Benefits		Costs	Costs	
	Healtcare savings	Other Morbidity	Tax revenue	Additional funding	VSL	Caregivers’ time	Consumer surplus	Profit	Net benefit
**$1.0 Tax**									
**After 10 years**	647	63	1720	4	654	39	1503	991	625
	(480–825)	(37–92)	(1492–1954)	(3–6)	(467–860)	(29–51)	(1305–1707)	(861–1126)	(292–987)
**After 35 years**	3604	305	3189	91	3534	218	2786	1838	6135
	(2668–4624)	(174–445)	(2717–3676)	(64–121)	(2521–4677)	(161–282)	(2376–3213)	(1567–2120)	(4209–8296)
**Lifetime**	4537	333	3402	199	3698	268	2972	1961	7105
	(3351–5817)	(190–485)	(2892–3925)	(142–265)	(2669–4850)	(198–347)	(2528–3430)	(1668–2263)	(4938–9506)
**$2.0 Tax**									
**After 10 years**	1153	112	2807	8	1168	70	2678	1925	700
	(867–1460)	(68–160)	(2436–3179)	(6–11)	(837–1529)	(52–90)	(2326–3038)	(1672–2184)	(142–1305)
**After 35 years**	6434	541	5209	162	6317	390	4970	3572	10187
	(4788–8180)	(322–776)	(4431–5984)	(116–214)	(4570–8241)	(289–500)	(4230–5712)	(3040–4106)	(6939–13760)
**Lifetime**	8105	591	5557	356	6612	481	5302	3811	11875
	(6009–10312)	(350–848)	(4708–6392)	(256–467)	(4785–8606)	(354–620)	(4503–6102)	(3237–4386)	(8101–15867)
**$3.0 Tax**									
**After 10 years**	1569	152	3395	11	1592	96	3597	2717	478
	(1172–1994)	(88–219)	(2959–3828)	(8–14)	(1136–2056)	(71–122)	(3136–4064)	(2369–3069)	(-303-1238)
**After 35 years**	8757	733	6299	221	8610	531	6675	5042	12994
	(6480–11169)	(421–1064)	(5381–7244)	(155–292)	(6113–11224)	(395–676)	(5703–7678)	(4308–5799)	(8415–17694)
**Lifetime**	11038	801	6721	486	9022	655	7122	5379	15250
	(8177–14062)	(459–1159)	(5733–7751)	(347–640)	(6451–11713)	(486–838)	(6076–8215)	(4589–6205)	(10090–20626)

Source/Note: SOURCE. Authors’ analyses based on structural models and an epidemiological model and inputs in Table A1.1. [2,9] NOTES: Numbers rounded up to single units. Information in 2014 Dollars and based on a discount rate of 4%. "Other Morbidity" includes savings for fewer disability pensions and fewer paid sick days and "Additional funding" includes costs for health care not attributable to overweight and obesity and public retirement pensions. VSL stands for value of statistical life. 95% sensitivity ranges in parentheses.

Appendix A5 in S1 File presents the estimated discounted net benefits under different assumptions around VSL. Compared to Fig 3, we see lower net benefits for the cohort’s lifetime (between USD 3.4 billion and USD 6.3 billion) and longer periods to achieve the break-even point under the conservative scenario of no VSL benefits. The opposite results hold under the optimistic scenario with full benefits from VSL, in which net benefits for the cohort’s lifetime are predicted to reach USD 8.1 billion for the one-MP tax and USD 17.6 billion for the three-MP tax. Across all scenarios in Appendix A5 in S1 File, we predict all SSB tax policies will yield positive net benefits.

## Discussion

We conducted a CBA of different SSB tax policies in Mexico and their implications for the government, consumers, and producers. We found that the initial costs in terms of profit and consumer surplus losses exceed benefits. Over time, healthcare savings, tax revenue, and VSL are the main contributors leading to positive net benefits. However, the time needed to reach the break-even point is inversely related to the SSB tax amount. All analyzed tax designs are predicted to reach net benefits within ten years. The government and consumers are the society’s members experiencing overall positive net benefits; however, consumers are expected to face initial losses due to costs (i.e., consumer surplus losses) surpassing benefits. These initial losses can be compensated through the value of public expenditures (e.g., education, pensions) from the collected tax revenue.

Our study contributes to the economic evaluations of SSB taxes through two main channels. First, our study represents a novel CBA based on the observed effectiveness of an existing SSB. Other studies, including consumers or producers, are based on ex-ante evidence of SSB tax policies [6,7,32]. Second, compared to previous SSB-tax evidence in Mexico restricted to the economic implications for the government [4], our study also includes consumers and producers. Moreover, we showed proportionally lower health and economic gains than SSB tax increases, which arises from the non-linear relationship between prices and demand in the demand model. Conversely, previous studies in Mexico assumed that SSB purchases would decrease by the same proportion as SSB taxes go up [3,4].

Our study has limitations. Our findings are not generalizable to the national level because the CBA is based on urban data. Evidence in rural Mexico showed lower SSB purchase reductions after the SSB tax implementation than in urban areas [33]. Thus, if we could implement our CBA in rural Mexico, it would be likely to find lower cost and benefit impacts. Another limitation corresponds to reporting results across the full urban population with no heterogeneous results across households. Low-income households would likely experience the highest health-related benefits due to previous evidence in urban Mexico showed these households experienced the largest SSB purchase reduction after the tax implementation [1,33]. Moreover, we might underestimate the tax benefits for three reasons. First, we only accounted for the indirect SSB effect on diabetes through BMI rather than modeling the direct effect too, as in other studies [3,34]. Second, we overlooked benefits linked to morbidity reductions or other outcomes such as oral health gains [35]. Third, our healthcare savings might represent a conservative estimate because some people get medical treatment in the private sector, which is more costly than publicly funded services [36,37]. Conversely, we did not account for tax implementation costs. However, these costs are expected to be negligible compared to SSB tax benefits (e.g., between 0.5 and 1.5% compared to tax revenue) [4,32]. Another limitation is that we did not account for any tax effect on employment. However, evidence shows no meaningful association between the SSB tax and employment rates in Mexico [38]. In terms of limitations for the structural model, this model did not allow us to account for the substitution between SSB and tap water in response to the tax implementation. However, this substitution likely plays a minor role because tap water is the main drinking water source for just about 10% of urban households in Mexico [39]. Due to the data limitations and the models’ assumptions, our results of the expected net benefit of the SSB tax should likely be taken as a conservative estimate.

## Conclusions

Our study contributes to SSB fiscal tax discussion in Mexico in several ways. We accounted for the fact that SSB taxes are not economically innocuous because they impose a burden on consumers and producers via consumer surplus and profit losses. Nonetheless, we showed that the existing one-MP SSB tax and higher SSB taxes are predicted to yield positive net benefits over time. However, the SSB tax displays a diminishing effect on SSB purchases. Consequently, population health improvements and healthcare savings will increase at a declining rate compared to the SSB tax. However, we also showed that the SSB tax policies in this study could generate significant economic benefits attached to health gains and tax revenue that will clearly surpass their costs. Therefore, an SSB tax policy in Mexico that best benefits society will depend on the interplay of the tax effect on health outcomes and economic consequences over time. Policymakers should consider the tradeoffs between costs and benefits to various stakeholders and time horizons to obtain health outcomes and healthcare savings. Future studies should extend the cost-benefit analysis of SSB taxes in Mexico by including sugar-density taxes considering their recent implementation in countries like South Africa, the United Kingdom, and Portugal.

## Supporting information

S1 File Supporting appendix. (DOCX)

S1 Data (XLSX)
