# Supplementary material for: Cost-benefit analysis of alternative tax policies on sugar-sweetened beverages in Mexico
Source: PLoS One. 2023 Oct 3;18(10):e0292276. doi: 10.1371/journal.pone.0292276 (PMC10547152; doi:10.1371/journal.pone.0292276)
Supplement: S1 File — (DOCX) [file pone.0292276.s001.docx]

**Cost-benefit analysis of alternative tax policies on sugar-sweetened beverages in Mexico**

**S1 File. Supporting appendix**

**Appendix A1 Inputs for the cost-benefit analysis**

**S1.1 Table. Sources and inputs for the cost-benefit analysis**

| **Source (alphabetically)** | **Input** |
| --- | --- |
| Banco de México [1] | - Exchange rate |
| Comisión Nacional de los Salarios Mínimos [2] | - Daily minimum wage |
| Fernández et al. (Economic Commission for Latin America and the Caribbean)[3] | - Health care treatment cost for kidney cancer and colon cancer in Chile and Ecuador - Calculated caregiving time by disease |
| Global Burden of Disease Collaborative Network[4] | - Information on incidence, prevalence, and case fatality by disease, sex, and age group |
| Global Food Research Program UNC [5] | - Kcal in sugar-sweetened beverages in Mexico |
| Hammit and Ibarraran[6] | - Estimate of the value of a statistical life |
| Mexican Ministry of Health[7] | - Health care treatment cost for diseases other than diabetes and kidney cancer - Days of employee absenteeism by disease - Wage information and proportion of people working by labor contract (formal-informal, with-without labor rights), sex, and age group the - Proportion of health expenditure devoted to health issues not associated with overweight and obesity |
| Mexican National Health and Nutrition Survey 2012[8] | - Body Mass Index by sex and age group - Distribution of consumption of sugar-sweetened beverages by sex and age group |

| **Source (alphabetically)** | **Input** |
| --- | --- |
| Mexican National of Statistics and Geography[9–11] | - Consumer price index - Money amount and proportion of people with a public retirement pension - Proportion of overall employment by sex and age group |
| Mexican National Population Council[12] | - Population size and overall mortality by sex and age group |
| Nielsen Mexico Consumer Panel Service[13] | - Purchase and price information in urban Mexico (>50,000 inhabitants) |
| Panadeiros and Pessino[14] | - Distribution of the average age-group per capita health expenditure |
| Salgado and Ng[15] | - Tax effect on purchases, prices, consumer surplus, and producer profit |
| Sanchez-Romero et al.[16] | - Health care treatment cost for diabetes |

**S1.2 Table. Main model inputs**

|  | **Mean** |  |  |
| --- | --- | --- | --- |
|  | **(Standard error)** | **Distribution** | **Reference** |
| PC-PD SSB Purchases (ml) | 200.37 | Normal | [8] |
|  | (9.55) |  |  |
| Body mass index | 28.46 | Lognormal | [8] |
|  | (0.06) |  |  |
| Relative risk of incidence | - | Lognormal | Table S2 in [17] based on [18,19] |
|  | - |  |  |
| **Economic inputs (2014 USD)** | **Point estimates** |  |  |
| **Healthcare costs** |  |  |  |
| Breast cancer | 30766.94 | - | [7] |
| Colon rectal | 26639.80 | - |  |
| Hypertensive heart disease | 3968.03 | - |  |
| Ischemic heart disease | 5113.96 | - |  |
| Kidney cancer^1^ | 19334.27 | - |  |
| Osteoarthritis | 104.68 | - |  |
| Stroke | 4280.40 | - |  |
| Diabetes |  | - |  |
| <55 years | 685.16 | - | [16] |
| 55-64 years | 817.13 | - |  |
| 65-74 years | 826.08 | - |  |
| 75-84 years | 1091.97 | - |  |
| 85+ years | 1101.62 | - |  |
| **Accompanying cost** |  | - |  |
| Breast cancer | 563.77 | - | [3] and [2] |
| Colon rectal | 550.36 | - |  |
| Diabetes | 19.21 | - |  |
| Hypertensive heart disease | 10.15 | - |  |
| Ischemic heart disease | 345.31 | - |  |
| Kidney cancer | 369.05 | - |  |
| Osteoarthritis | 244.24 | - |  |
| Stroke | 458.99 | - |  |
| **Other economic inputs** |  |  |  |
| Healthcare costs for non-obesity needs | 257.58 | - | [7] and [14] |
| Expected labor income | 790.31 | - | [7] and [10] |
| Expected public retirement pensions | 255.16 | - | [9] |
| Expected disability pension | 11.46 | - | [7] |
| Value of statistical life | 280743.21 | - | [6] |
| Exchange rate (one Mexican Peso in USD) | 0.08 | - | [1] |
| Note: SSB: Sugar-sweetened beverages. PC-PD: Per capita per day. ^1^Information for breast cancer is only for women. We calculated the healthcare cost for kidney cancer using the disease cost ratio described in the methods section. For public retirement pensions, disability pensions, and labor income (which we used to calculate paid sick days), we present these values as expected values depending on the respective probability of getting a pension or being employed across all age-sex groups. | | | |

**S1.3 Table. Summary statistics of the purchase and price information for the structural model in the** **Mexican** **non-dairy and non-alcoholic beverage market.**

|  |  | 2012-2013 (pre-tax) | 2014-2015 (post-tax) |
| --- | --- | --- | --- |
| Market shares (%) | Taxed beverages | 19.61 | 17.56 |
|  | Untaxed beverages | 20.27 | 18.19 |
|  | Outside option | 60.11 | 64.25 |
| PC-PD purchases (ml) | Taxed beverages | 182.77 | 165.42 |
|  |  | (10.50) | (9.81) |
|  | Untaxed beverages | 189.05 | 171.68 |
|  |  | (12.66) | (13.93) |
|  | Outside option | 561.25 | 608.49 |
|  |  | (42.53) | (64.22) |
| Prices per liter (Mexican pesos) | Taxed beverages | 8.52 | 9.40 |
|  |  | (1.52) | (1.44) |
|  | Untaxed beverages | 2.04 | 2.05 |
|  |  | (1.92) | (2.04) |
|  | Observations | 696 | 696 |
| *Note:* Table extracted from Salgado JC and Ng SW. Simulating international tax designs on sugar-sweetened beverages in Mexico. *PLOS ONE*. 2021[15]. The outside option mainly corresponds to bottled plain water provided by local producers. Prices are quantity-weighted average prices in terms of real values in January 2014. Standard deviation in parentheses. PC-PD: Per capita Per day. Salgado and Ng [15] calculated these summary statistics based on data from Nielsen through its Mexico Consumer Panel Service (CPS) for the food and beverage categories for January 2012 – December 2015. The Nielsen Company, 2016. Nielsen is not responsible for and had no role in preparing the results reported herein. | | | |

**S1.4 Table. Average body mass index by sex and age group in urban Mexico**

| Age group | Men | Women |
| --- | --- | --- |
| 20 – 24 years | 25.97 | 25.86 |
| 25 – 29 years | 27.31 | 27.56 |
| 30 – 34 years | 28.17 | 28.73 |
| 35 – 39 years | 28.94 | 29.54 |
| 40 – 44 years | 29.15 | 30.01 |
| 45 – 49 years | 28.88 | 30.33 |
| 50 – 54 years | 29.00 | 30.80 |
| 55 – 59 years | 28.07 | 30.55 |
| 60 – 64 years | 27.94 | 30.06 |
| 65 – 69 years | 27.48 | 29.52 |
| 70 – 74 years | 27.62 | 29.00 |
| 75 – 79 years | 26.56 | 28.14 |
| ≥ 80 years | 25.45 | 26.60 |
| Source: Mexican National Health and Nutrition Survey 2012[8] | | |

**S1.5 Table. Variation of the average per capita per day consumption of sugar-sweetened beverages by sex and group compared to the overall average in urban Mexico (average reference = 100)**

| Age group | Men | Women |
| --- | --- | --- |
| 20 – 30 years old | 149.3 | 127.4 |
| 31 – 50 years old | 158.6 | 67.4 |
| 51 - 70 years old | 97.6 | 39.1 |
| 71 years old and older | 65.5 | 63.3 |
| Source: Mexican National Health and Nutrition Survey 2012[8]  Notes: Average reference is based on information on SSB consumption from all Mexicans residing in urban areas | | |

###

### **Appendix A2. Tax effect on purchases, consumer surplus, producer profits, and tax revenue**

Based on the structural model of the Mexican SSB market[15], we estimate the effect of the tax of one Mexican peso (MP) per liter of sugar-sweetened beverage (SSB) on purchases in post-tax years (i.e., 2014- 2015) as in equation (A2.1):

|  | $\Delta Purchases=\sum_{t}^{T} Q_{t}\sum_{j=1}^{J} [S_{jt}\left( \tilde{p}_{t}^{\mathrm{NoTx}}, X; \theta\right)- S_{jt}\left( \tilde{p}_{t}^{\$1Tx}, X; \theta\right)];$ | (A2.1) |
| --- | --- | --- |

where $\Delta Purchases$ is the overall change in purchases in liters, $Q_{t}$ is the market size in terms of overall purchases of both taxed and untaxed beverages of interest, and $S_{jt}\left( \tilde{p}_{t}^{\mathrm{NoTx}}, X; \theta\right)$ and $S_{jt}\left( \tilde{p}_{t}^{\$1Tx}, X; \theta\right)$ are the estimated market shares for beverage $j\in(1,\ldots,J)$ in the absence or presence of the one-MP tax, respectively. Thus, $\tilde{p}_{t}^{\mathrm{NoTx}}$ stands for the simulated price vector with no tax and $\tilde{p}_{t}^{\$1Tx}$ stands for the simulated price vector under the one-MP tax. $X$represents the set of beverages’ observable characteristics other than prices (e.g., sugar content), and $\theta$ is the estimated vector of structural parameters from the demand model.

According to the functional form of the demand model (i.e., random-coefficients logit model), the tax effect on consumer surplus for household $i$ is estimated using equation (A2.2):

|  | $\Delta_{i}=\ln\sum_{t\in T,j\in J} e^{u_{ijt}\left( \tilde{p}_{t}^{\mathrm{NoTx}},X,\theta\right)} -\ln\sum_{t\in T,j\in J} e^{u_{ijt}\left( \tilde{p}_{t}^{\$1Tx},X,\theta\right)}$ | (A2.2) |
| --- | --- | --- |

Where $u_{ijt}(.)$ is the indirect utility function for household $i$ from beverage $j$ at the market-month $t$. The demand model is built upon this utility function. To bring the tax effect on consumer surplus to monetary terms, we use the parameter $\alpha_{i}$ from the demand model that captures the household $i$’s marginal utility linked to income. Thus, the change in consumer surplus ($\Delta CS$) in monetary terms across all households is:

|  | $\Delta CS= Q\left[ \frac{1}{H}\sum_{i=1}^{H} \frac{1}{\left\vert\alpha_{i} \right\vert}\Delta_{i} \right]$ | (A2.3) |
| --- | --- | --- |

The profit change for multi-product producer $f\in(1,\ldots,F)$ is:

|  | $\Delta Profit=\sum_{t}^{T} \sum_{f=1}^{F} \left[ \pi_{f}\left( \tilde{p}_{t}^{\mathrm{NoTx}},X,\theta\right)- \pi_{f}\left( \tilde{p}_{t}^{\$1Tx},X,\theta\right) \right]$ | (A2.4) |
| --- | --- | --- |

where $\pi_{f}\left( \tilde{p}_{t}^{\mathrm{NoTx}},X,\theta\right)$ and $\pi_{f}\left( \tilde{p}_{t}^{\$1Tx},X,\theta\right)$ stand for producer $f$’s profit in the absence or presence of the one-MP tax. This producer gets this profit by selling both taxed and untaxed beverages.

The change in welfare ($\Delta Welfare)$ in monetary terms in urban Mexico under the one-MP tax is calculated as in equation (A2.5):

|  | $\Delta Welfare=\frac{\left( \Delta CS+\Delta Profit \right)}{Tax Revenue}$ | (A2.5) |
| --- | --- | --- |

where $Tax Revenue$ is the money collected by the government from SSB purchases, which arises from the volume of SSB purchases in liters and the SSB tax per liter.

In a similar fashion as described above, we calculate the effect for the alternative tax amounts by substituting $\tilde{p}_{t}^{\$1Tx}$ for $\tilde{p}_{t}^{\$2Tx}$ (simulated price vector under the tax of two MP per SSB liter) or $\tilde{p}_{t}^{\$3Tx}$ (simulated price vector under the tax of three MP per SSB liter).

**Appendix A3. Health care treatment costs not attributable to overweight and obesity**

In 2014, according to the Mexican Ministry of Health, 34% of the national public health expenditure was allocated to treat health issues attributable to overweight and obesity [7]. To calculate the annual health care treatment cost of a death averted due to the SSB tax, we subtract from the 2014 per capita public health expenditure the proportion of 34% attributable to overweight and obesity following the approach in this regard by the National Institute of Public Health (Ana Basto Abreu, researcher, personal communication, May 8, 2019). The resulting outcome is what we consider as the health care treatment cost not attributable to overweight and obesity.

To account for the expected heterogeneity in health care treatment cost by age, we use the estimates by Panadeiros and Pessino of the cost-related dispersion by age group with respect to per capita public health expenditure as shown in S3.1 Exhibit [14]

**S3.1 Table. Mexico: average age-group per capita health expenditure by the public health care system and the social security system (average reference = 100)**

| Age group | Compared to the average reference |
| --- | --- |
| 0-4 years | 52.4 |
| 5-9 years | 30.8 |
| 10-14 years | 24.0 |
| 14-19 years | 36.6 |
| 20-24 years | 60.5 |
| 25-29 years | 75.2 |
| 30-34 years | 72.1 |
| 35-39 years | 67.7 |
| 40-44 years | 73.5 |
| 45-49 years | 84.0 |
| 50-54 years | 95.3 |
| 55-59 years | 111.4 |
| 60-64 years | 125.9 |
| 65-69 years | 144.9 |
| 70-74 years | 166.0 |
| 75-79 years | 194.5 |
| $\geq$80 years | 193.5 |
| Source: Table extracted from Panadeiros M, Pessino C.Consecuencias fiscales del envejecimiento poblacional. Proyecciones agregadas del gasto en salud para 10 países de América Latina. *Doc PARA DISCUSIÓN N^o^ IDB-DP-601*. 2018.[14] | |

Depending on the person’s age when his/her death was averted due to the SSB tax or as this person ages over time, we multiply the measure of the treatment cost not attributable to overweight and obesity by the corresponding value in S3.1 Exhibit. The resulting value is the variable ${ATC noOO}_{a}$, which enters equation (A4.4) below.

### **Appendix A4. Cost-Benefit Analysis**

The economic benefits for the government due to fewer prevalent cases of disease attributable to the SSB tax are composed of savings for a lower demand for health care treatment, fewer disability pensions, and fewer paid sick days as shown in equations (A4.1), (A4.2), and (A4.3), respectively.

|  | $Public Treatment Saving=\sum_{y=1}^{Y_{a}} \sum_{s=1}^{S} \sum_{a=1}^{A} \left[ \frac{{Morbidity}_{sa}*{ATC}_{s}}{{(1+r)}^{y}} \right]_{y}$ | (A4.1) |
| --- | --- | --- |

where $y\in(1,\ldots,Y_{a})$ is each year after the tax implementation until the last year with available health-related information for the age group $a\in(1,\ldots,A)$, and $s\in(1,\ldots,S)$ stands for the diseases of interest for age group $a$. Age group $a$ includes both women and men for the relevant age group. ${Morbidity}_{sa}$ is the reduction in prevalent cases of disease $s$ for age group $a$ (compared to the reference population in the epidemiological model) and ${ATC}_{s}$ is their associated average treatment costs. The reduction in prevalent cases captures the changes in incidence and mortality induced by the tax implementation. Future costs are discounted at a rate $r$. For our analyses, we set a discount rate equivalent to 4%.

|  | $Public Disability Pension Saving=\sum_{y=1}^{Y_{a}} \sum_{s=1}^{S} \sum_{a=1}^{A} \left[ \frac{{Morbidity}_{sa}*PrDPension{*DPension}_{a}}{{(1+r)}^{y}} \right]_{y}$ | (A4.2) |
| --- | --- | --- |

Where $PrDPension$ is the probability of holding a public disability pension and ${DPension}_{a}$ is the annual disability pension for a person in the age group $a$.

| $Paid Sick Days Savings=\sum_{y=1}^{Y_{a}} \sum_{s=1}^{S} \sum_{a=1}^{A} \left[ \frac{{Morbidity}_{sa}*{(Abse}_{s}/365)*\sum_{w=1}^{w=2} {Work}_{wa}*I_{wa}}{{(1+r)}^{y}} \right]_{y}$ | (A4.3) |
| --- | --- |

Where ${Abse}_{s}$ is the number of days of employee absenteeism per year due to disease $s$ for age group $a$. This information on ${Abse}_{s}$ was based on the study on the economic cost attributable to the obesity in Mexico by the Mexican Ministry of Health, where it was assumed 180 days of employee absenteeism per year for any cancer and 20 days for other diseases [7]. 365 stands for the number of days within a year. ${Work}_{wa}$ is the probability of working under the labor contract $w$, which provides labor benefits for age group $a$ whose annual salary is $I_{wa}$. We considered two types of labor contracts: formal jobs with labor benefits and informal jobs with labor benefit.^[[1]](#footnote-1)^

By reducing the number of premature deaths, the SSB tax implementation will cause additional costs for the government by people who live longer and thus continue their utilization of the social security system. This utilization is composed of health care treatment for reasons not attributable to overweight nor obesity and public retirement pension for people aged $\geq$ 65 years. Equations (A4.4) and (A4.5) represent each of these components, respectively.

|  | $Public Treatment Costs=\sum_{y=1}^{Y_{a}} \sum_{a=1}^{A} \left[ \frac{{Additional Life Years}_{a}*{ATC noOO}_{a}}{{(1+r)}^{y}} \right]_{y}$ | (A4.4) |
| --- | --- | --- |

|  | $Retirement Pension Cost=\sum_{y=1}^{Y_{a}} \sum_{a=1}^{A} \left[ \frac{{Additional Life Years}_{a}*PrRPension*{RPension}_{a}}{{(1+r)}^{y}} \right]_{y}$ | (A4.5) |
| --- | --- | --- |

Where ${Additional Life Years}_{a}$ is the additional annual years of life for age group $a$ attributable to the SSB tax, ${ATC noOO}_{a}$ is the average annual treatment cost for issues not attributable to overweight and obesity, $PrRPension$ is the probability of getting a public retirement pension from the government, and ${RPension}_{a}$ is the sum money of this pension. $PrRPension$ equals zero for people in age group $a$ not older than 65 years.

Variables related to probabilities and amounts of public retirement pensions, public disability pensions, and labor income can vary by sex across age groups. In the supplementary metadata, we present the expected values of these variables (i.e., probability times the average amount) by specific sex-age groups. In the empirical application of our cost-benefit analysis, we accounted for the difference in these variables by age-sex groups.

We estimated the economic savings for the government after the tax implementation using equation (A4.6)

|  | $Government Saving=\left( Public Treat Saving+ Public Disability Pension Saving+Paid Sick Days Savings \right)-(Public Treat Costs + Retirement Pension Cost)$ | (A4.6) |
| --- | --- | --- |

The welfare loss among consumers and producers due to the SSB purchase decrease attributable to the SSB tax is:

|  | $SSB Purchase consumer and producer Welfare Loss=\sum_{y=1}^{Y_{a}} \sum_{a=1}^{A} \left[ \frac{{SSB Purchase}_{a}*TxAmount*WL per Tx}{{(1+r)}^{y}} \right]_{y}$ | (A4.7) |
| --- | --- | --- |

where ${SSB Purchase}_{a}$ is the annual overall SSB purchases for age group $a$ measured in liters when the SSB tax is into effect, $TxAmount$ is the tax amount per liter of SSB, and $WL per Tx$ is the ratio of the reduction in profits and consumer surplus per MP collected as tax revenue.

We used equation (A4.8) to account for the economic benefits of the health improvements attributable to the SSB tax from the consumer perspective:

|  | $Aggregate VSL=\sum_{y=1}^{Y_{a}} \sum_{a=1}^{A} \left[ \frac{{Mortality}_{a}*VSL*(100-Health Internalization)}{{(1+r)}^{y}} \right]_{y}$ | (A4.8) |
| --- | --- | --- |

where ${Mortality}_{a}$ is the annual number of saved lives by the SSB tax for age group $a$, $VSL$ is our preferred estimate of the value of statistical life, and $Health Internalization$ corresponds to the degree to which people internalize the negative consequences on health attached to SSB consumption, which we set to be equal to 20% as explained in the paper’s section of methods.

In addition to the economic value of health benefits for SSB consumers as in equation (A4.8), the SSB tax will also benefit their relatives who serve as caregivers due to fewer prevalent cases of disease as shown in equation (A4.9):

|  | $Caregiving Savings= \sum_{y=1}^{Y_{a}} \sum_{s=1}^{S} \sum_{a=1}^{A} \left[ \frac{{Morbidity}_{sa}*time cost*({time}_{sa})}{{(1+r)}^{y}} \right]_{y}$ | (A4.9) |
| --- | --- | --- |

where $time cost$ is equal to the minimum wage per hour and ${time}_{sa}$ is the average total time spent by patients’ relatives during the health care treatment and recovery due to disease $s$ for patients in cohort $a$. We derived information on ${time}_{sa}$ from Fernandez et al. [3]. Specifically, we calculated ${time}_{sa}$ as the ratio between the overall cost linked to caregiving time in Mexico and the multiplication between the cases of the relevant diseases and the average hourly minimum wage in Mexico [2,3], assuming people work eight hours a day. We calculated ${time}_{sa}$ by disease and sex group.

The net economic benefit of the SSB tax results from the differences between costs and benefits, as shown in equation (A410):

|  | $NetBenTx=Benefits-Costs$  where  $Benefits=Government Saving+Aggregate VSL+Caregiving Savings+TxRev$  $Cost= SSB Purchase Consumer and Producer Welfare Losses$ | (A4.10) |
| --- | --- | --- |

and $TxRev$ is the collected tax revenue and is calculated using equation (A4.7) by substituting $WL per Tx$ for the tax amount per SSB liter.

### **Appendix A4. Tax effect on health outcomes**

**S4.1 Table.** Effect by SSB tax policy on avoided new incident cases

|  | **Diabetes** | **IHD** | **Stroke** | **Cancer** | **HtHD** | **Osteoarthritis** |
| --- | --- | --- | --- | --- | --- | --- |
| **$1.0 MP Tax** |  |  |  |  |  |  |
| **After 10 years** | 85112 | 7922 | 8881 | 1730 | 3210 | 9388 |
|  | (43844-127456) | (5132-11213) | (4524-13583) | (1268-2272) | (1646-5003) | (6481-12559) |
| **After 35 years** | 232964 | 31372 | 34032 | 7003 | 16437 | 32968 |
|  | (119605-355722) | (19307-45714) | (16524-53216) | (4921-9364) | (8089-26168) | (22223-44951) |
| **Lifetime** | 244973 | 36185 | 42181 | 9463 | 29031 | 38690 |
|  | (125654-374914) | (17141-58595) | (17156-69420) | (5886-13548) | (12613-48982) | (25132-53749) |
| **$2.0 MP Tax** |  |  |  |  |  |  |
| **After 10 years** | 150786 | 14148 | 15888 | 3111 | 5719 | 17021 |
|  | (80320-221132) | (8756-19711) | (8316-23948) | (2285-4018) | (2819-9095) | (11765-23081) |
| **After 35 years** | 415450 | 56112 | 60987 | 12599 | 29283 | 59926 |
|  | (219417-621404) | (32810-80433) | (30660-94050) | (8888-16781) | (13654-47453) | (40575-82708) |
| **Lifetime** | 437623 | 64556 | 75564 | 16998 | 51631 | 70330 |
|  | (232975-655026) | (27291-104999) | (32896-121905) | (10654-24097) | (20698-88833) | (46935-97371) |
| **$3.0 MP Tax** |  |  |  |  |  |  |
| **After 10 years** | 204098 | 19327 | 21742 | 4239 | 7727 | 23031 |
|  | (108073-304766) | (12297-27014) | (11878-32192) | (3084-5526) | (4072-11981) | (15962-30808) |
| **After 35 years** | 564419 | 76800 | 83606 | 17177 | 39564 | 81067 |
|  | (297657-860110) | (46953-111362) | (42991-126580) | (12019-22975) | (20169-62497) | (54317-110270) |
| **Lifetime** | 595356 | 88878 | 103813 | 23198 | 69736 | 95148 |
|  | (313341-905399) | (40432-142727) | (45655-164823) | (14365-33347) | (31747-116893) | (62854-131389) |
| Source/Note: SOURCE. Authors' analyses based on structural models and an epidemiological model and inputs in Table A1.1. NOTES. Numbers rounded up to single units. IHD: ischemic heart disease; HtDH: hypertensive heart disease; and cancer represents a set of cancers (breast cancer, colon cancer, and kidney cancer). 95% sensitivity ranges in parentheses. | | | | | | |

**S.4.2 Table.** Effect by SSB tax policy on avoided deaths

|  | **Diabetes** | **IHD** | **Stroke** | **Cancer** | **HtHD** |
| --- | --- | --- | --- | --- | --- |
| **$1.0 MP Tax** |  |  |  |  |  |
| **After 10 years** | 1053 | 1170 | 1045 | 410 | 324 |
|  | (531-1636) | (701-1730) | (512-1637) | (285-557) | (166-511) |
| **After 35 years** | 18445 | 11478 | 11176 | 3411 | 3556 |
|  | (9115-28702) | (6776-17175) | (5126-17818) | (2320-4701) | (1726-5702) |
| **Lifetime** | 50240 | 18168 | 21517 | 5491 | 9020 |
|  | (23831-78356) | (6865-31788) | (7368-36520) | (3190-8177) | (3601-15405) |
| **$2.0 MP Tax** |  |  |  |  |  |
| **After 10 years** | 1874 | 2089 | 1871 | 739 | 577 |
|  | (970-2834) | (1195-3067) | (955-2896) | (514-992) | (274-925) |
| **After 35 years** | 32926 | 20525 | 20036 | 6150 | 6342 |
|  | (16732-50186) | (11399-30115) | (9682-31634) | (4186-8287) | (2782-10391) |
| **Lifetime** | 89916 | 32443 | 38610 | 9905 | 16078 |
|  | (44899-136777) | (10738-55885) | (14682-64881) | (5824-14547) | (5834-28104) |
| **$3.0 MP Tax** |  |  |  |  |  |
| **After 10 years** | 2541 | 2858 | 2563 | 1007 | 778 |
|  | (1310-3898) | (1664-4202) | (1344-3861) | (703-1368) | (401-1221) |
| **After 35 years** | 44722 | 28105 | 27484 | 8382 | 8560 |
|  | (22602-69022) | (16119-42046) | (13727-42359) | (5768-11448) | (4148-13615) |
| **Lifetime** | 122327 | 44765 | 53127 | 13520 | 21706 |
|  | (60656-189192) | (15939-77504) | (20957-87709) | (7761-20126) | (9133-36873) |
| Source/Note: SOURCE. Authors' analyses based on structural models and an epidemiological model and inputs in Table A1.1. NOTES. Numbers rounded up to single units. IHD: ischemic heart disease; HtDH: hypertensive heart disease; and cancer represents a set of cancers (breast cancer, colon cancer, and kidney cancer). 95% sensitivity ranges in parentheses. | | | | | |

### **Appendix A5. Cumulative discounted net benefits by scenarios of the value of statistical life**

**S5.1 Fig.**  Cumulative discounted net benefit by SSB tax policy

| 1. Conservative scenario (no benefit from value of statistical life)   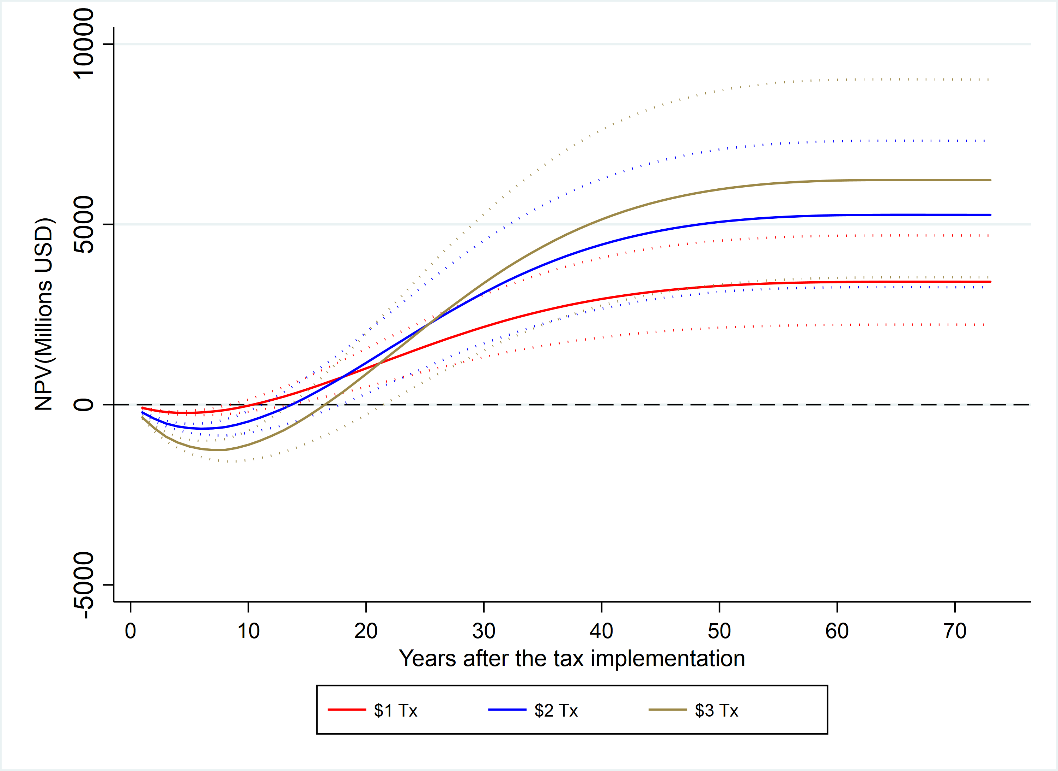 | 1. Optimistic scenario (full benefit from value of statistical life)   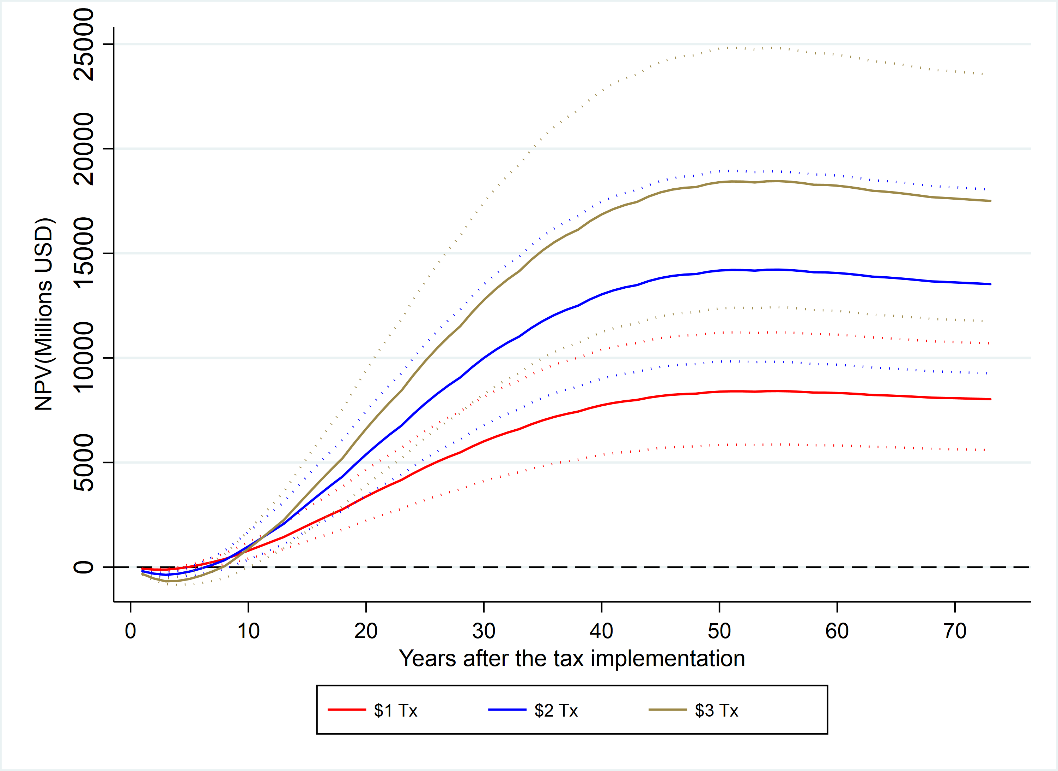 |
| --- | --- |
| Source/Note: SOURCE: Authors' analyses based on structural models and an epidemiological model and inputs in Table A1.1. NOTES: Information in 2014 Dollars and based on a discount rate of 4%. NPV stands for net present value. Sensitivity ranges (95%) in dotted lines | |

**References**

1. Banco de Mexico. Tipo de cambio para solventar obligaciones denominadas en dólares de los EE.UU.A., Para solventar obligaciones. [cited 3 Nov 2019]. Available: http://www.anterior.banxico.org.mx/portal-mercado-cambiario/

2. Comisión Nacional de los Salarios Mínimos. Nuevos salarios mínimos 2014, por área geográfica generales y profesionales . [cited 25 Jan 2019]. Available: http://www.conasami.gob.mx/nvos_sal_2014.html

3. Fernández A, Martínez R, Carrasco I, Palma A. [The cost of the double burden of malnutrition: Social and economic impact Summary of the pilot study in Chile, Ecuador and Mexico]. 2017.

4. Global Burden of Disease Collaborative Network. Global Burden of Disease Study 2017 (GBD 2017) Results. In: Seattle, United States: Institute for Health Metrics and Evaluation (IHME) [Internet]. 2018 [cited 8 Aug 2019]. Available: http://ghdx.healthdata.org/gbd-results-tool

5. University of North Carolina Global Food Research Program. UNC Mexican Nutrition Fact Panel. 2019.

6. Hammitt JK, Ibarrarían ME. The economic value of fatal and non-fatal occupational risks in Mexico City using actuarial- and perceived-risk estimates. Health Econ. 2006. doi:10.1002/hec.1137

7. Unidad de Análisis Económico de la Secretaría de Salud. Impacto Económico del Sobrepeso y la Obesidad en México 1999-2023. Nota Técnica. 2015.

8. Gutiérrez J, Rivera-Dommarco J, Shamah-Levy T, Villalpando-Hernández S, Franco A, Cuevas-Nasu L, et al. Encuesta Nacional de Salud y Nutrición 2012. Resultados Nacionales. Cuernavaca, México: Instituto Nacional de Salud Pública; 2012.

9. Instituto Nacional de Estadística y Geografía. [How and How Many Mexicans Have Access to Pensions]. In: Comisión Nacional del Sistema de Ahorro para el Retiro Blog [Internet]. 2018 [cited 21 Apr 2019]. Available: https://www.gob.mx/consar/articulos/quienes-y-cuantos-mexicanos-tienen-acceso-a-una-pension

10. Instituto Nacional de Estadística y Geografía. [National Survey of Ocupation and Employment, population 15 y and older]. 2014 [cited 19 Apr 2019]. Available: https://www.inegi.org.mx/programas/enoe/15ymas/default.html#Tabulados

11. Instituto Nacional de Estadística y Geografía. [National Consumer Price Index]. 2015 [cited 13 Jun 2019]. Available: https://www.inegi.org.mx/temas/inpc/

12. Consejo Nacional de Población. [Population Projections 2010-2050]. 2014 [cited 1 May 2017]. Available: http://www.conapo.gob.mx/es/CONAPO/Proyecciones

13. The Nielsen Company. Mexico Consumer Panel Service (CPS) for the food and beverage categories for January 2012 – December 2015. 2016.

14. Panadeiros M, Pessino C. Consecuencias fiscales del envejecimiento poblacional. Proyecciones agregadas del gasto en salud para 10 países de América Latina. DOCUMENTO PARA DISCUSIÓN N^o^ IDB-DP-601. 2018.

15. Salgado Hernández JC, Ng SW. Simulating international tax designs on sugar-sweetened beverages in Mexico. Paraje G, editor. PLoS One. 2021;16: e0253748. doi:10.1371/journal.pone.0253748

16. Sánchez-Romero LM, Penko J, Coxson PG, Fernández A, Mason A, Moran AE, et al. Projected Impact of Mexico’s Sugar-Sweetened Beverage Tax Policy on Diabetes and Cardiovascular Disease: A Modeling Study. PLoS Med. 2016. doi:10.1371/journal.pmed.1002158

17. Veerman JL, Sacks G, Antonopoulos N, Martin J. The impact of a tax on sugar-sweetened beverages on health and health care costs: A modelling study. PLoS One. 2016. doi:10.1371/journal.pone.0151460

18. Asia Pacific Cohort Studies Collaboration, Ni Mhurchu C, Parag V, Nakamura M, Patel A, Rodgers A, et al. Body mass index and risk of diabetes mellitus in the Asia-Pacific region. Asia Pac J Clin Nutr. 2006;15: 127–33.

19. James WPT, Jackson-Leach R, Mhurchu CN, Kalamara E, Shayeghi M, Rigby NJ, et al. World Health Organization Report Part Title: Overweight and obesity (high body mass index) Report Part Title: Comparative Quantification of Health Risks Report Subtitle: Global and Regional Burden of Disease Attributable to Selected Major Risk Factors Report Editor(s). World Health Organization. 2004.

1. The savings for fewer paid sick days for people with this right and who are part of the informal sector are not a benefit for the government. However, we included these savings in equation (A4.3) to avoid presenting this equation twice (i.e., one for the government and another for the employers in the informal job sector). Moreover, these savings in the informal sector represent less than 2% of the overall savings for fewer paid sick days. An example of people with informal jobs with labor benefits could be domestic employees. [↑](#footnote-ref-1)
